# Urinary Deposits; Their Diagnosis, Pathology, and Therapeutical Indications

**Published:** 1847-01

**Authors:** 


ART. XI..—*Urinary Deposits; their Diagnosis, Pathology, and Therapeutical Indications.* By GOLDING BIRD, A.M., M.D., F.R.S., &c. Second Edition.—*London,* 1846. 8vo, pp. 356.

IT is only eighteen months since we gave an analysis of the first edition of this valuable treatise (Brit. and For. Med. Rev., July, 1845). The rapid sale of the work has confirmed the justness of our high opinion of it, as there expressed. So short a period has elapsed since the book was first published that the author has found little room for the addition of new matter; but it is evident that this edition has been carefully revised and improved in various particulars. It is a book which ought to be in every medical library.

